# Supplementary material for: Light-directed biofilm formation reveals the functional contributions of periplasmic cytochromes to the electrochemical activity of Shewanella oneidensis
Source: mBio. 2026 Jul 16;17(8):e01264-26. doi: 10.1128/mbio.01264-26 (PMC13463773; doi:10.1128/mbio.01264-26)
Supplement: Supplementary file 1 [file mbio.01264-26-s0001.pdf]

Supplementary Information for

Light-directed Biofilm Formation Reveals the Functional Contributions of Periplasmic Cytochromes to the Electrochemical Activity of *Shewanella oneidensis*

Fengjie Zhao[1#], Christina M. Niman[1#], Marko S. Chavez[1,2], Joshua T. Atkinson[1,2,3], Bridget E. Conley[4], Jeffrey A. Gralnick[4], James Q. Boedicker[1,5]*, and Mohamed Y. El-Naggar[1,5,6]*

[1]Department of Physics and Astronomy, University of Southern California, Los Angeles, California 90089, United States
[2]Omenn-Darling Bioengineering Institute, Princeton University, Princeton, New Jersey 08540, United States
[3]Department of Civil and Environmental Engineering, Princeton University, Princeton, New Jersey 08540, United States
[4]BioTechnology Institute and Department of Plant and Microbial Biology, University of Minnesota–Twin Cities, St. Paul, Minnesota 55108, United States
[5]Department of Biological Sciences, University of Southern California, Los Angeles, California 90089, United States
[6]Department of Chemistry, University of Southern California, Los Angeles, California 90089, United States

[#] Fengjie Zhao and Christina M. Niman contributed equally to this work
*Correspondence to: James Q. Boedicker, boedicke@usc.edu
Mohamed Y. El-Naggar, mnaggar@usc.edu

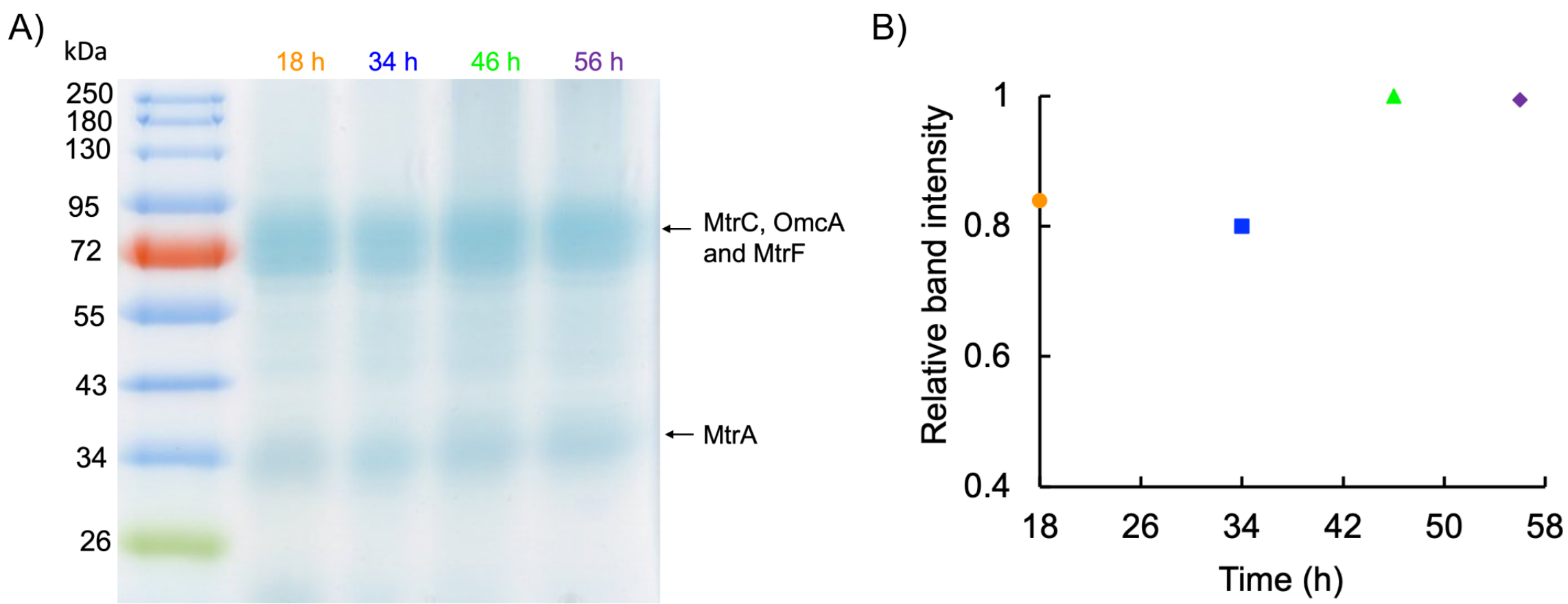

Figure S1. Cytochrome expression of the triple deficient mutant Δ*cctA*Δ*fccA*Δ*nrfA* at different time points of the iron reduction measurements. (A) TMBZ heme stain SDS-PAGE protein gel of Δ*cctA*Δ*fccA*Δ*nrfA* at different time points. (B) Band intensity of cytochrome MtrA at different time points. 18 h and 34 h are the time points during the lag phase of the iron reduction. 46 h and 56 h are the time points after the lag phase of the iron reduction.

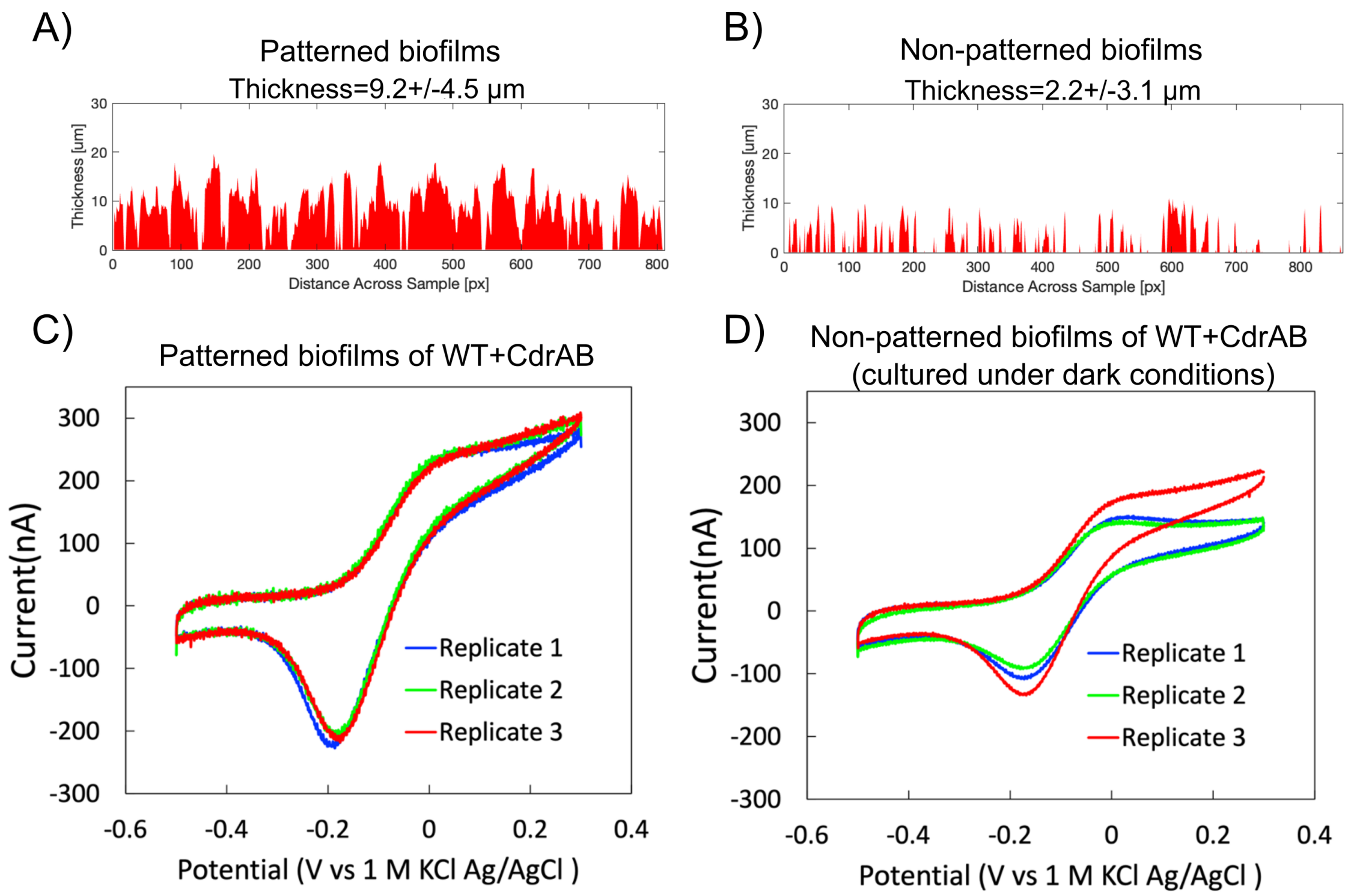

Figure S2. Biofilm thickness and cyclic voltammetry measurements of blue-light patterned biofilms and non-patterned native biofilms. Biofilm thickness of patterned biofilms (A) and non-patterned biofilms (B), measured from the cross-sectional confocal images based on the method used in our previous work (1). The patterned biofilms, with an average thickness of ~ 10 µm, are more uniform and thicker than the non-patterned biofilms with an average thickness of ~ 2 µm. Cyclic voltammetry measurements of patterned biofilms (C) and non-patterned biofilms (D). Patterned biofilms gave more consistent currents across different replicates than those of the non-patterned biofilms.

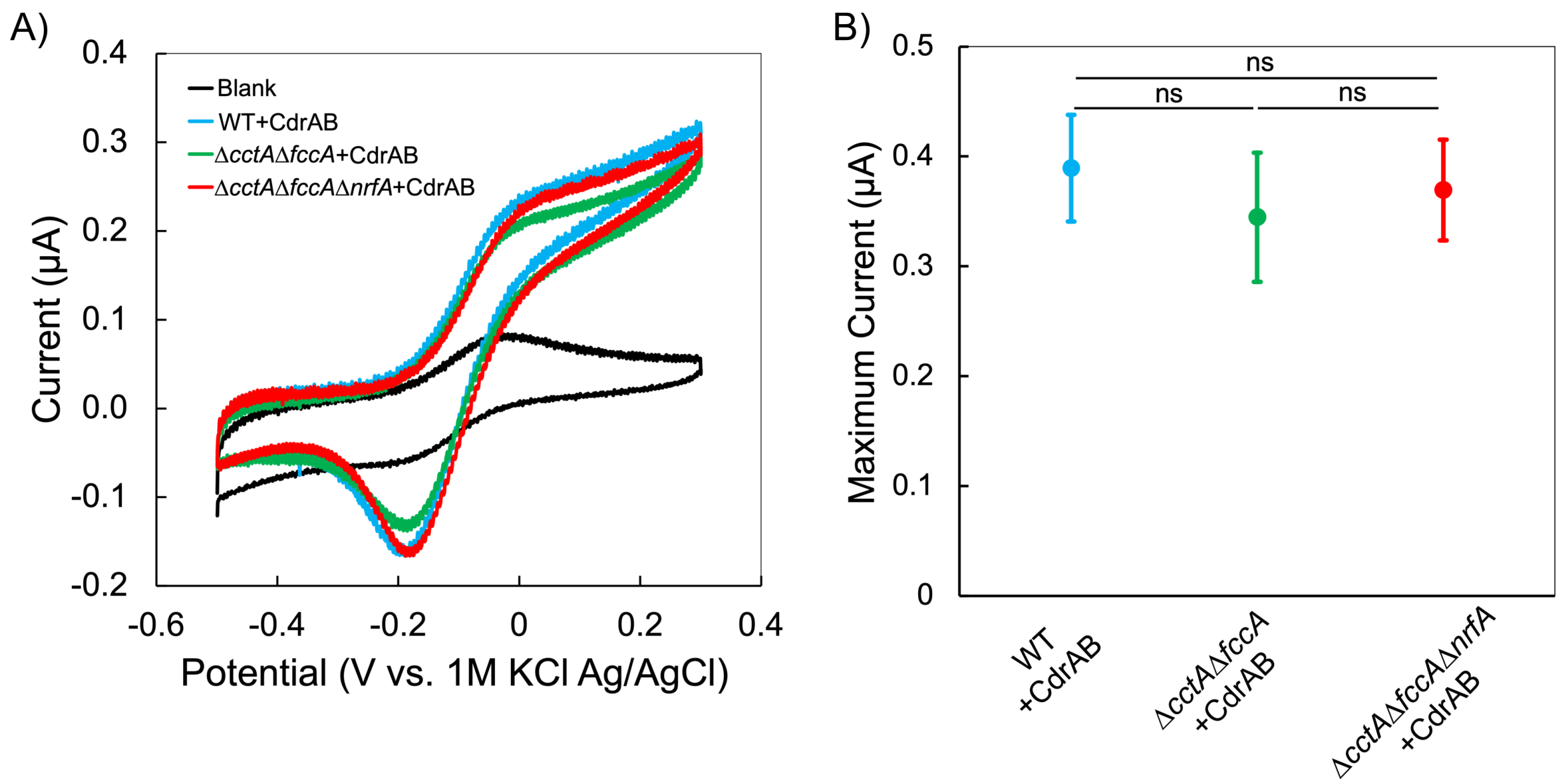

Figure S3. Cyclic voltammetry measurements of WT+CdrAB, Δ*cctA*Δ*fccA*+CdrAB, and Δ*cctA*Δ*fccA*Δ*nrfA*+CdrAB patterned biofilms without adding flavins. (A) Representative cyclic voltammograms of blue light patterned biofilms of WT+CdrAB, Δ*cctA*Δ*fccA*+CdrAB, and Δ*cctA*Δ*fccA*Δ*nrfA*+CdrAB. (B) The maximum currents from the cyclic voltammograms for each mutant. For quantitative comparison, the maximum current of each voltammogram was collected. The means and standard deviations were calculated from multiple biological replicates: 11 for the WT+CdrAB, 4 for the Δ*cctA*Δ*fccA*+CdrAB, and 5 for the Δ*cctA*Δ*fccA*Δ*nrfA*+CdrAB. The maximum current is 0.39 ± 0.05 μA for the WT+CdrAB, 0.34 ± 0.06 μA for the Δ*cctA*Δ*fccA*+CdrAB and 0.37 ± 0.05 μA for the Δ*cctA*Δ*fccA*Δ*nrfA*+CdrAB. No significant differences in maximum currents from the cyclic voltammograms were observed among the three patterned biofilms (two-tailed unpaired *t*-test, $p = 0.1594$ for WT+CdrAB vs. Δ*cctA*Δ*fccA*+CdrAB, $p = 0.4547$ for WT+CdrAB vs. Δ*cctA*Δ*fccA*Δ*nrfA*+CdrAB, and $p = 0.5003$ for Δ*cctA*Δ*fccA*+CdrAB vs. Δ*cctA*Δ*fccA*Δ*nrfA*+CdrAB). Significance is indicated as ns (not significant, $p > 0.05$).

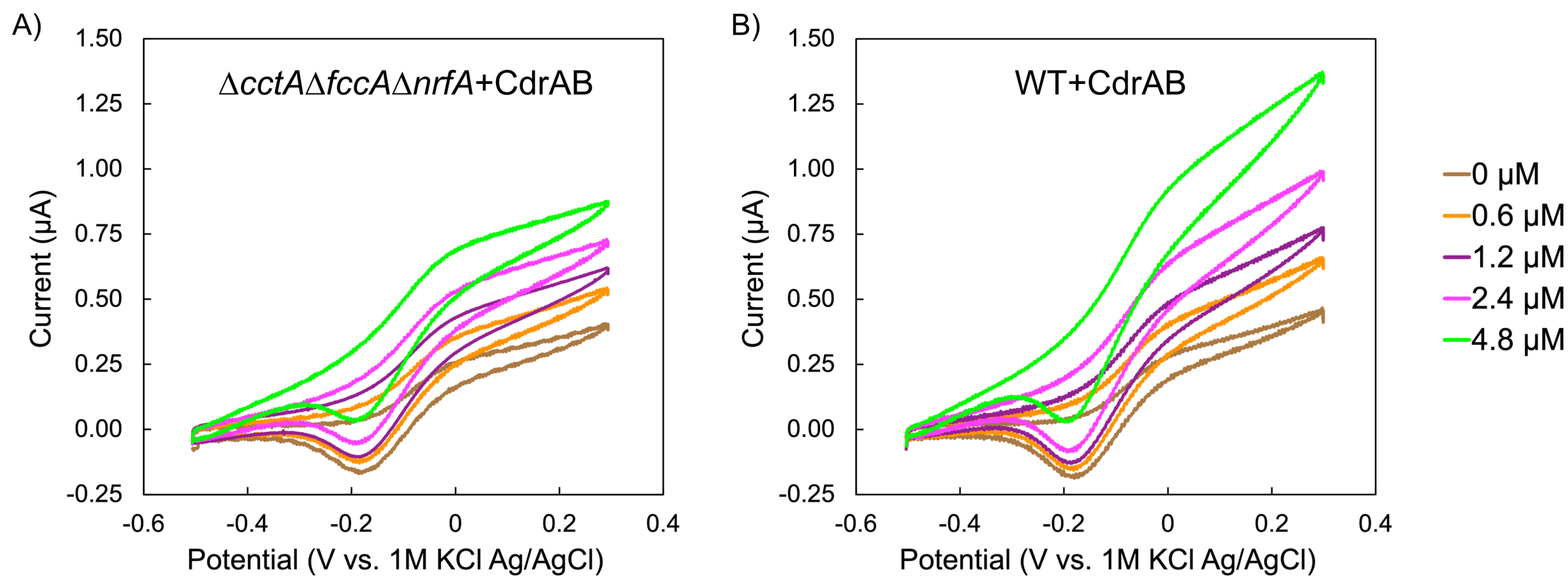

Figure S4. Representative cyclic voltammetry measurements of Δ*cctA*Δ*fccA*Δ*nrfA*+CdrAB (A) and WT+CdrAB (B) patterned biofilms with different concentrations of flavin mononucleotide (FMN) added.

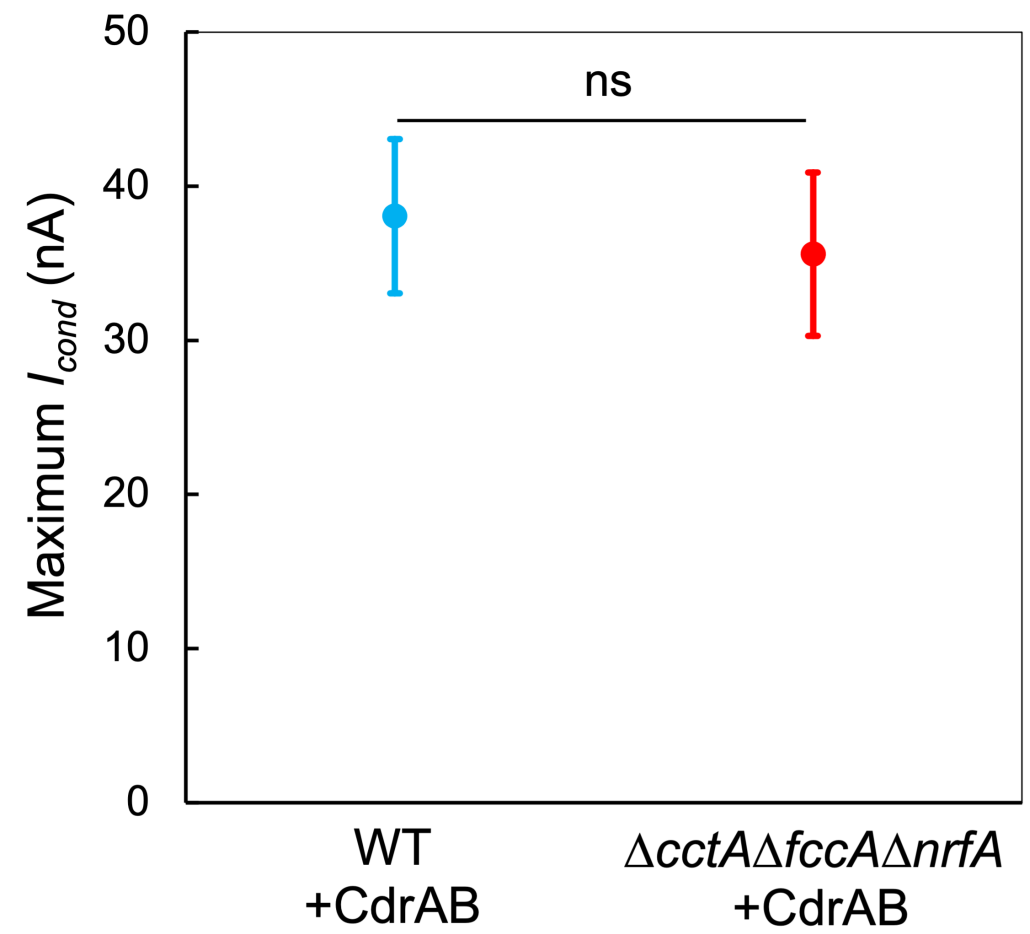

Figure S5. Maximum conduction currents of WT+CdrAB and Δ*cctA*Δ*fccA*Δ*nrfA*+CdrAB patterned biofilms from electrochemical gating measurements. The data (mean ± SD) were obtained from three biological replicates. The maximum conduction current is 38.04 ± 5.29 nA for the WT+CdrAB and 35.60 ± 5.30 nA for the Δ*cctA*Δ*fccA*Δ*nrfA*+CdrAB. No significant difference in maximum conduction current was observed between patterned WT+CdrAB biofilms and Δ*cctA*Δ*fccA*Δ*nrfA*+CdrAB biofilms (two-tailed unpaired *t*-test, $p = 0.6021$). Significance is indicated as ns (not significant, $p > 0.05$).

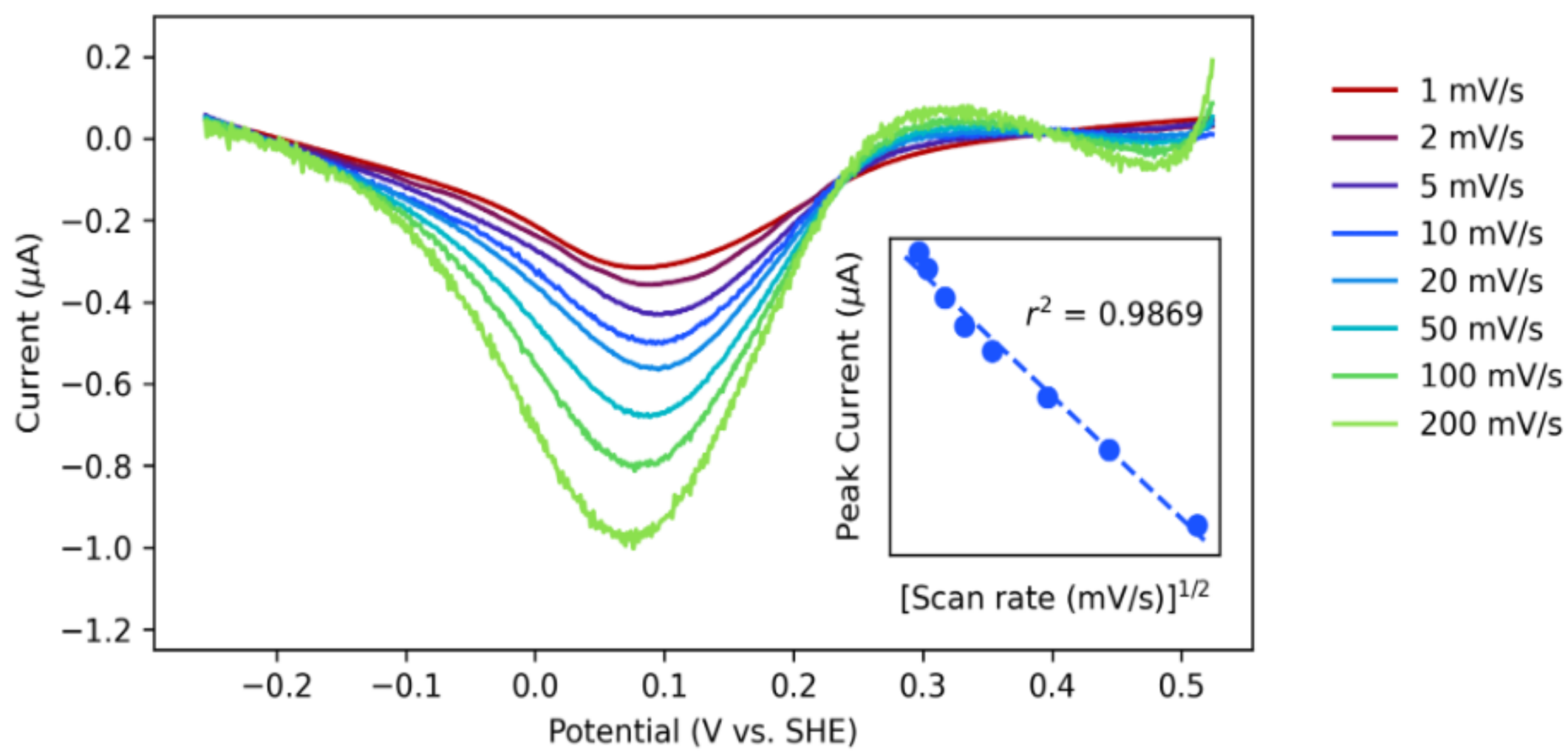

Figure S6. Scan rate dependent non-turnover cyclic voltammetry on WT+CdrAB biofilms patterned onto IDAs. Inset figure shows the peak currents observed in the data increase with increasing scan rate and clearly scale linearly with the square root of the scan rate.

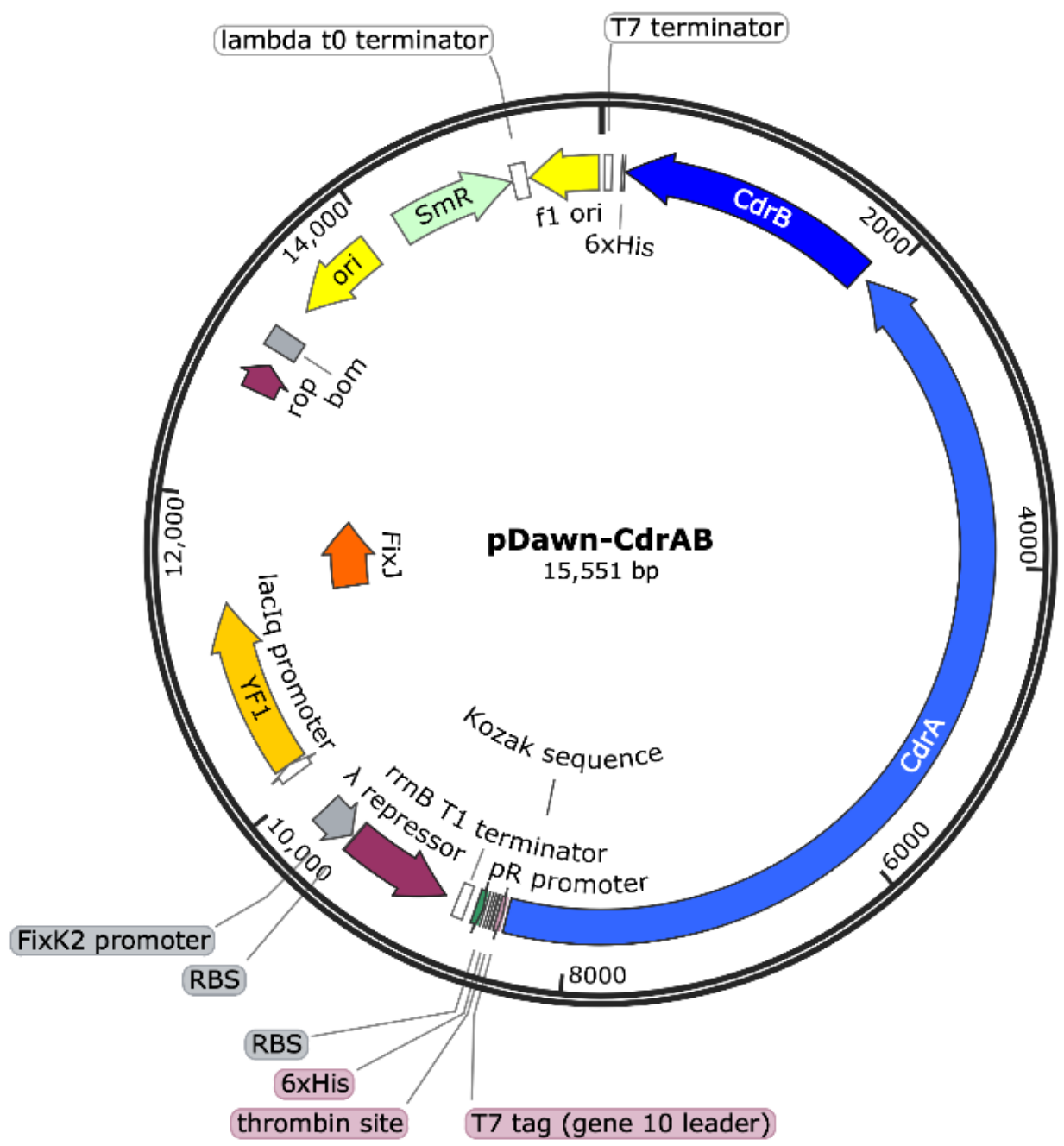

Figure S7. Plasmid map of pDawn-CdrAB.

Table S1. Strains and plasmids used in this study.

Strain	Description	Source
WT	*Shewanella oneidensis* MR-1, wild type strain	Lab stock
Δ*cctA*Δ*fccA* (JG3107)	MR-1 derivative without genes encoding periplasmic cytochromes CctA and FccA	(2)
Δ*cctA*Δ*fccA*Δ*nrfA* (JG4347)	MR-1 derivative without genes encoding periplasmic cytochromes CctA, FccA, and NrfA	This work
WT+CdrAB	MR-1 with pDawn-CdrAB	(1)
Δ*cctA*Δ*fccA*+CdrAB	JG3107 with pDawn-CdrAB	This work
Δ*cctA*Δ*fccA*Δ*nrfA*+CdrAB	JG4347 with pDawn-CdrAB	This work
**Plasmids**	**Description**	**Source**
pDawn-CdrAB	Expression of CdrAB in response to blue light using the pDawn genetic circuit	(1)

Table S2. Full DNA sequence of plasmid pDawn-CdrAB

```
ATCCGGATATAGTTCCTCCTTTCAGCAAAAAACCCCTCAAGACCCGTTTAGAGGCCCCAAGGGGTTATGCTAGTTATTGCTC
AGCGGTGGCAGCAGCCAACTCAGCTTCCTTTCGGGCTTTGTTAGCAGCCGGATCTCAGTGGTGGTGGTGGTGGTGCTCGA
GTCAGAAGCGCGCCACCACGTTGAACAGGACCCGCGTCTCGCGCTTCCCATCGCGCTGGTCGGCTGGCTCGCCGATGACC
TGGCGGGCAATCTCCGGGTTGAACGACAACTGGTCGGTGAGGTTGAAGCGCACCCCGGCACCGATCGAGCCGAGCTTCTG
CTGCTCGTCATCGTCCACGTCGAGGTTGTAGGTCTTGCCCCAGTCGTAGAAAGCGTAGGCCTGGTAGCTGCGCAGCCAGC
TATAGCCCACCGCCTGGCCATAGCGCGGTTCGAGCTTCAGGGCGAAGCCACGGTCACCGGTGATGTTGCCCGGGGCGAA
GCCGCGACCGATCCCGCTGCCGCCGACGCCGAAGCGCTCGCTCGGCAGCAACGGGTCGAAGCTGTACTGCCATTGCAATT
CGCCATACACCGACCAGGGTCCGCCGAGCCCTTGCAGACGCCCGATGTTGCCGTTGAGATAGACGAAACGTCCGTCGGCC
ACTTCGCGGGAACGACGTTCATCGTCCTTCTTGGTCGCGCCCCACATGTCCAGGCCGATGTTGTTGCGCACCTCCGCCAGG
TTGATGCCCATCCAGCGGTCGACCCAGTCATAGGTCAGGCGTTGCTGGAACACCCGCACATGGTCACGACCGGGGTTCGA
GGCGTCGCTGGCGAAGTCGCGCAGGCGGGTCTCGGTATCCTTCCACTGCAACGCCAGGCCGCCGCTCAGGTTCTGCGAG
CGCGAGCGGATGAAGGGATGGTTGTAGGAAATGCTGCCTTCGGTGCTCTTGCTGACGCTGTCGAAGGGGAATACCGAGTC
CGGGATGTCCGGGTTGACCTTCGAATAGGAACCCATGATCTGCAGGGTATCGCCGGCGAACATGCCACCCAGCGGCAACG
TGCCGCGCAACTGGATGTAGCGGGTGGCATCGCTGAACGGCGAGATCGCGCCGAGCACGTCGATCTGCTCGTAGAAGCC
CAGGCGGGAGAACTCGGTGGCGCCGAAGGCCAGGGTGGTCGGGTCGGTGTAGTGCGAGCCGCGGTTGTCGAGGCTGAC
GAAGCCTTCGTATCGCTGCTGTTGCAAGGCCACGTCGAGGTTCGACGAGCCCAGCGCCTGACCCGGGCTGAGCACGCTCT
GCACGTTGACCCCCGGCAGGTCGCCGACCAGCAGCAGGCCGCGCTCCAGGGTATCGGCATCCAGCGGACGTTTTTCGGT
CAGCGGCGCGAGCAGTTCCTCCACTCGCACCCGCGGCCCATCGACGGTTCCCTTGATCTGCACGTGTTCGAGGTAACCCT
CGATCACCTGCAGGCGTACCTTGCCGCCCTCGATGCTCTGCTCCGGCACCACTACCTGGGTCAACAGGAAGCCGTCCTGG
CGGTACTTGGCGGTGAGGCGGTTGGCCAGGTCGACCAGTTCGGCCAGGGACACCTGCTCGCCGAGCATATGCTGGTAATA
GGACTCGAGCACCGCCTTGTCATAGCGGGTGTTGCCTTCGATGCGTATCTCGCGCAGTTTGAAGCGGATCTGTTCGCTGCC
TTGCGGCGCGACCTGCTTGGGCACCACCAGCGGCGCCTCGGGCTTGGCTTGCGGCTCGCTGCGGGGCTTGAAGCGTTCA
TCGAGCTGGGATGGCTGTACAGTCCCTGGCAACTCCACGGCGGCGGCGGAAGCAGCCAGCATCCCGCAGCCACAGAGCG
TCACCCAGCGAAGCGTCCATTGCGAAGGTTTCATCATTCACCTCGGGCAGATGACGCACTACCGCCCGGAGACGATAGTGC
GCATTGCGAAAAGGGTTAGAAATTCAGAGGCTTGTTGAAGCAGCTCCAGTAGTCGGCCAGGTAGCTCTGGTTCGAGCAACC
CGCCCCACTCTCGGCCTCTGCCCTGAGCGGACGATCGGTGAACTGCCCCACCCGCTTGGCGCTGGCGTCCTCCGCCGGA
CCCGCGGCGGTAGCCGGCAGCAGGTTAGGATCGCTGACGTCCATGCCCAGAGCGTAGGCGCGCAGGAACGGGTTTTCGA
GGAACGGGTTTTCCAGCGTGGTGGTGCGGTAGACCGCATCCCCGGGGGTATCCGGATCGCGCCCCTTCTGCAGTTCGCGC
AAGTCGGAGAGCGCTGCCAGTTGCCCCAGGTCGCCCGGCGTGACCGGCAGCGGCAGGATGCTCAGCAGGTTGCCCTGGT
AGTTGAGCTGGTAGTTGCCGCTGGTGAGGGCAAGCCCACCCTGGAGGATGTCGTACTGGCCTACGGCTTCGCCGCCCACC
CGACCGAGCCCGCCGGCGAGCACCTGACCGGCGCTATCGCCATTCTTCAGGCCGGAAACCTGATAGGTCAGCGCCGGGT
CGGCGGTGCCGACCTGCTTGCTCTTGGCATCGGCGAATACGTTGAGCAGCGCCTTGGTGATGGTCAGGTCATTACCCTGG
```

TAGGCCAGGTCATAGTTGCCACTGGTCAGCACCAGGCCGCCCTGGTTGATGCCATACACACCCACGTTCTCGCCTGCGTCG
CGGTTCAGCCCGCCGGTCAGGATCGAGCCGGCGCTGTCACCGTTCTTCAGGCCGCTGACCTGGTAGGTCAGGCTCGGGT
CAGCGTCGCCATAGACCTTGGTCTTGCCGTCGGCGATCACATTGAGCAGCGCCTTGGTGATGGTCAGGTTGTTGCCCTGGT
AAGCCAGGTCATAGTTACCGCTGACCAGGCCCAGTCCACCCTGGTTGATGCCGTACACGCCGACATTCTCGCCAGCCACG
CGGCTCAGGCTGCCACCGTTGAGCACCGCGCCAGCGGTATCACCGTTCTTCAGGCCGCTGACCTGGTAGGTCAGGCTCGG
ATCGGCATCGCCGTAGACCTTGGTCTTGGCGTCGGCGATCACATTCAGCAGTGCCTTGGTGATGGTCAGGTTGTTGCCCTG
GTAAGCCAGGTCATAGTTACCGCTGACCAGACCCAGGCCACCCTGCTGAATCGCATAGTTGCCTACGTTTTCGCCGGAGAC
ACGAACCAGGCCGCCGCCATTGAGCACCGCGCCAGCGGTATCGCCATTCTTCAGGCCGCTGACCTGGTAGGTCAGGCTCG
GGTCGGCATCGCCATAGACCTTGGTCTTGGCATCGGCGATCACATTCAGCAGTGCCTTGGTGATGGTCAGGTTATTGCCCT
GGTAGCTCAGGTCGTAGTTACCGCTGTTCAATGCCAGGTCGCCCTGGTTGATGCCATACACACCCACGTTCTCGCCTGCGG
CGCGGTTCAGCCCGCCGGTCAGGATCGAACCGGCGCTGTCGCCGTTCTTCAGGCCGCTGACCTGGTAGGTCAGGCTCGG
ATCGGCATCGCCGTAGACCTTGGTCTTGGCATCAGCGATCACATTCAGCAGTGCCTTGGTGATGGTCAGGTTGTTGCCCTG
GTAAGCCAGGTCATAGTTACCGCTGACCAGACCCAGGCCACCCTGCTGAATCGCATAGTTGCCTACGTTTTCGCCGGAGAC
ACGAACCAGGCCGCCGCCATTGAGCACCGCGCCAGCGGTATCGCCATTCTTCAGGCCGCTGACCTGGTAGGTCAGGCTCG
GATCGGCATCGCCGTAGACCTTGGTCTTGGCGTCAGCGATCACATTCAGCAGCGCCTTGGTGATGGTCAGGTTATTGCCCT
GGTAGCTCAGGTCGTAGTTACCGCTGTTCAATGCCAGGTCGCCCTGGTTGATGCCGTACACGCCGACATTCTCGCCAGCCA
CGCGGCTCAGGCTGCCACCGTTGAGCACCGCGCCAGCGGTATCACCGTTCTTCAGGCCGCTGACCTGGTAGGTCAGGCTC
GGATCGGCATCGCCGTAGACCTTGGTCTTGGCGTCGGCGATCACATTCAGCAGTGCCTTGGTGATGGTCAGGTTGTTGCCC
TGGTAAGCCAGGTCATAGTTACCGCTGACCAGACCCAGGCCACCCTGCTGAATCGCATAGTTGCCTACGTTTTCGCCGGAG
ACACGAACCAGGCCGCCGCCATTGAGCACCGCGCCAGCGGTATCGCCATTCTTCAGGCCGCTGACCTGGTAGGTCAGGCT
CGGGTCGGCATCGCCATAGACCTTGGTCTTGGCATCGGCGATCACATTCAGCAGTGCCTTGGTGATGGTCAGGTTATTGCC
CTGGTAGCTCAGGTCGTAGTTACCGCTGTTCAATGCCAGGTCGCCCTGGTTGATGCCATACACACCCACGTTCTCGCCTGC
GGCGCGGTTCAGCCCGCCGGTCAGGATCGAACCGGCGCTGTCGCCGTTCTTCAGGCCGCTGACCTGGTAGGTCAGGCTC
GGATCGGCATCGCCGTAGACCTTGGTCTTGGCATCAGCGATCACATTCAGCAGTGCCTTGGTGATGGTCAGGTTGTTGCCC
TGGTAAGCCAGGTCATAGTTACCGCTGACCAGACCCAGGCCACCCTGCTGAATCGCATAGTTGCCTACGTTTTCGCCGGAG
ACACGAACCAGGCCGCCGCCATTGAGCACCGCGCCAGCGGTATCGCCATTCTTCAGGCCGCTGACCTGGTAGGTCAGGCT
CGGATCGGCATCGCCGTAGACCTTGGTCTTGGCGTCGGCGATCACATTCAGCAGTGCCTTGGTGATGGTCAGGTTATTGCC
CTGGTAGCTCAGGTCGTAGTTACCGCTGTTCAATGCCAGGTCGCCCTGGTTGATGCCATACACACCCACGTTCTCGCCTGC
GGCGCGGTTCAACCCACCGGTCAGGATCGAACCGGCGGTATCACCGTTCTTCAGGCCGCTGACCTGGTAGGTCAGGCTCG
GATCGGCATCGCCGTAGACCTTGGTCTTGGCGTCGGCGATCACATTCAGCAGCGCCTTGGTGATGGTCAGGTTATTGCCCT
GGTAGCTCAGGTCGTAGTTCGCGCTGAGCAGGCCCAGTCCACCCTGGTTGATGCCGTACACGCCGACATTCTCGCCAGCC
ACGCGGCTCAGGCTGCCGCCGTTGAGCACGGCACCGGCGGTGTCGCCGTTCTTCAGCCCGGACACCTGGTAGGTCAGTG
CCGGATCGGCATCGCCATAGACCTTGGTCTTGGCATCGGCGATCACATTGAGCAGTGCCTTGGTGATGGTCAGGTTATTGC
CCTGGTAGTTCAGGGTGTAATTGCTGCTGACCAGGCCCAGTCCACCCTGGTTGATGCCGTACACGCCGACGTTCTCGCCAG
CCACGCGGCTCAGGCTGCCGCCGTTGAGCACGGCACCGGCGGTATCGCCGCGCTTGAGACCGGAGACCTGGTAGGTCAG
CGCCGGATCCAGGTCGCCGTAGACCTTGGTCTTGGCATCGGCGATCACGTTCAGTTGTGCCGGGGTGATGCGCAGGTCGT
TGCCGACGTAGCTGAGGGTGTAATTGGCCGTGTTGAGGCCCAGCCCGCCCTGGAGTATCCCGTAGTTGCCGACGTTCTCC
CCGGCCACCCGGCCGAGGTTGCCGCTCAGCACGCCAGCGGCGGTATCGCTGTTCTTCAGGCCACTGACCTGGTAGGTGA
GGGTCGGATCGGCGTCGCCATAGACCTTGGTCTTGGCGTCGGCGCTGACGATCAATTGCGCCGGCGTGACGGTCAGCCTG
CCGTCCTGGCGCACCAGTTGGTAACGCTGGCTGAAATACTCGCTGCCATTGGCGTTACCGGTGATCGCGTACTGCCCGACA
TTCGACGCCTGGGTCGCCGTGGTGCTGGCGGTCGCCTGGTAGAGGTCGTTGAACTGACTGTTGACGTAGGCGTCGATGGC
GTTGATGCCGGACATCGTGGCCGTCAGAGTCGGATTCACATCACCGTAGACGCGGGTAGCGTCATTGGCGGTCAGGGTGG
CGGTCTGCTGGCGGGAAAGGATGTCGCCGGCAGTGGTGGTGGTCTTGAACATCTCCACCTGGGCGCGCTCGGCGAAGCT
GTTGAACGCTCCCTCGCGCATGAACAGGTCCAGGCGGGTATCGACGCGGCTGGTCTTGCCGCCGAGGCCGAAGATCAGTT
CCTCGGTCAGGCCGGTGCCGACGTCGTACTGCAGCTCGTCGGTCACGCCGATCACGCCGCCGAAGCCCTTGACGCCCCA
ACTGGTGCCCCAGTTTCCGGTCCAGGTGGTGACGCTGCCGTTGCCGGCGGCGACGGTGAACCCGCTGGGCGCACCGCCG
TTCGGGGTGGCGGTGACGTTGCTGCCGTTGACCTGGTAGCGCAGGTTGGTCACCAGGTAGTCCGACGAGTCGTTGGTAGG
GAAACTGGCGGAACCGCTGACCCGCCGATAGGGCAGCGCGGTGAACACCAGGCGGTCGAACTCGCTGTTGCCGTCGGCA
GTCAGCGTGTAGTTGCCGGTGGTGCCAACGCTGTTGGCCTCGAGAATCCGCGTACCGACGCTGGAGACGATGTTCTGCGC
GGTGCCGGTCATGCTCTGGTTGATGAACGGATCGAACACCGTGCCGCTCGTGGTCACGCCGGTGGCGTTGCTCTGCCGGT
ACTTGAGCGTACCGTTGAAGGTCTGCAGCGCCAGGTTGCCGGCGTAGTTGTCCCAGGAAGCCAGGTCGATGCTACCAGCA
CGGCCGGCGAGGTTGGCGGCGATCAGCGCGGTACCGGCCTGCTGGCTGATCTTGCCGGCGGTGCTGGTCTGCATCCACA
GGGTGCCGCTGCCCAGGTCGACGATGCCGCTGGTCTGCGGCGTGCCGCCGCTGAGGAAATTGATGTTGCCGCCGGCGCG
GATTGCCAGGCTGCCGTTGCGGAACAGGATCGGCGCAGAGATATCCACCGCACCGGCAGGTCCGGTGGCGATCAGCTCC
AGGCCGCGACTGGCGCCGCCCAGGTTGCTGGTGATCAGCGGCGCTTCCACCGACAGCCGGTCGCTGGCGCTGAGGGTCA
CCTTGCCGCCTGCCAGCGCACCGGTAACCACCGAGGCATTCACCGTCGCGTCGCCGCTGGCGCCGTTGGCGCCCCCCAC
CGAGCCGGAGGTGCCGCCGGAGGCGACGATGCGCAGGGTGGTCGGGTCGAGCAGCCAGGTGCCACCGTTGCCGTAGGG
TGCGGCGACGCTGACGTAGGCGCTGTCGGCGATGCTCAGGTTGACCTTGCCGGAGGTCTCCACCAGGCCGCCGTTGCCG
CCCTGGGCGCCGCCGCGCGCGAGGATCCGCCCGTCCATGCGGGTATTGCCGTCGGACCAGACCACCGCCAGGCCGCCAT
TGCCCTTGCCGGTAGCCGAGACGTCGATGCTGGCGCCCTTGGCCAGCGTAGCGTTCTTCGCCCGTGGCAGCTCGCCGCG
GCCCTGGAAGTCGCCGCCGATGCGCACGCTGCCGCCCTGTGCGGTGCCGCGGGCGTCGATGCTGGCGGTGCTCGCCAG
GGCCACCTGCTGCCCGCTGACCAGCACGTCGCCGCCGTTCTTCGGACCGCTGACGTCGATCTTGCCGGCGATCTGGATGT
CCCCGGACTTGCTTACCAGCGCCACGCTGCCCTCGCGCACCTCGGTGCCGCGGGCAAGGATCACGCCGGACAGGTTGAC
CGCGTTGTCGACCACCCGGGAAGCCTGCTCGGCGGTGATCAGCACGCTACCGCCGTCGGCCTGGATCGCGCCACCGTTG
CTGACCAGCGCCTGGGCATCCTGCGGCTTGCGGGTAACCGGGTCGGTCACGGCGATCGACACCAGGCCATCGCCGTAGA
GGTCGATGGTCGCGGCATTGCCGGCGGCCATCGCCACGGTGCCGAGGCGGGCGTTGATCACGCCGTTGTTGGCCACGGA
CGGCGCGACGAAGGCCGCCAGGCCTTTCTCCGCGACGCTGATGCTGCCGTCGTTGACGATCCGCGCGTCGGGGTTTCCG
GGCTGGTCGAAGATCAGCTTGCCCTGGCGGAAGTGTTCGTCGCTGATGTTCGCCGTGGTGGTGATCAGGGAGCCCACGTC
GACCTTGGCCGAGCCGCTGAAGACCACGCCGTTGGAGTTGTAGATGATCACCTGGCCGGGCGCGGAGATGCGCCCGGCG
ATCACGCTTTCCTGGCCTGCGGTGACCCGGTTCAGGGTCGACGAGGTCACACCGGGCTGGTTGAAGCGCACCGCCTCGTC

GGCGGAGACGTCGAAGCCCTTCCAGTTGATGATCGCGCGCTGGGTGCTCTGGTCGATCAGCGTGGTCTTGCCCTGCTGGC
TGATCTTGATCTCGTTGGGGTTGAACTGGGCACCGGTAGGTGCCGCCATGGCCGCGTTGATGCTGGCGATGGCGAACATG
AGGGTGCTGCTGGCGGCAAGGACGAAACGCCCTTCCCTACCAGCTTTCGAAGAGGTGCGCTTGCCGCGCTGGCTATCCGG
ACGGACCATTCTAGTAGGTTTCCTGTGTGAGGATCCGACCCATTTGCTGTCCACCAGTCATGCTAGCCATATGGCTGCCGC
GCGGCACCAGGCCGCTGCTGTGATGATGATGATGATGGCTGCTGCCCATGGTATATCTCCTTCTTAAAGTTAAACAAAATTA
TTTCTAGAGCAACCATTATCACCGCCAGAGGTAAAATAGTCAACACGCACGGTGTTACTCTAGTATATAAACGCAGAAAGGC
CCACCCGAAGGTGAGCCAGTGTGACTCTAGTAGAGAGCGTTCACCGACAAACAACAGATAAAACGAAAGGCCCAGTCTTTC
GACTGAGCCTTTCGTTTTATTTGATGCCTGGCTCTAGTAGCGATCTACACTAGCACTATCAGCGTTATTAAGCTACTAAAGCG
TAGTTTTCGTCGTTTGCAGCGCCAAACGTCTCTTCAGGCCACTGACTAGCGATAACTTTCCCCACAACGGAACAACTCTCAT
TGCATGGGATCATTGGGTACTGTGGGTTTAGTGGTTGTAAAAACACCTGACCGCTATCCCTGATCAGTTTCTTGAAGGTAAA
CTCATCACCCCCAAGTCTGGCTATGCAGAAATCACCTGGCTCAACAGCCTGCTCAGGGTCAACGAGAATTAACATTCCGTCA
GGAAAGCTCGGCTTGGAGCCTGTTGGTGCGGTCATGGAATTACCTTCAACCTCAAGCCAGAATGCAGAATCACTGGCTTTTT
TGGTTGTGCTTACCCATCTCTCCGCATCACCTTTGGTAAAGGTTCTAAGCTCAGGTGAGAACATCCCTGCCTGAACATGAGA
AAAAACAGGGTACTCATACTCACTTCTAAGTGACGGCTGCATACTAACCGCTTCATACATCTCGTAGATTTCTCTGGCGATTG
AAGGGCTAAATTCTTCAACGCTAACTTTGAGAATTTTTGCAAGCAATGCGGCGTTATAAGCATTTAATGCATTGATGCCATTA
AATAAAGCACCAACGCCTGACTGCCCCATCCCCATCTTGTCTGCGACAGATTCCTGGGATAAGCCAAGTTCATTTTTCTTTTT
TTCATAAATTGCTTTAAGGCGACGTGCGTCCTCAAGCTGCTCTTGTGTTAATGGTTTCTTTTTTGTGCTCATCTAGTATTTCTC
CTCTTTTCTAGACTCCGTTGTGATGACGCATTGGTACGCGGTATCGGGAGGTTCGAAAATTTCGAGCGATATCTTAAGGGGG
GTGCCTTACGTAGAACCCCGTAGGTCATGCCCGAGGCCGGTCCTGGATGGCGCGGCGGATACGCTTGAGCAGGTTTTCGT
CGAGAAGCGGCTTCAAAACCACGTCTTTTACGCCGGCCTCGGCGGCCCGGGTCGAGATGTTTTCGTCCGGATAGCCGGTG
ATCAGGATCACGGGCGTAGATCTCGATCCTCTACGCCGGACGCATCGTGGCCGGCATCACCGGCGCCACAGGTGCGGTTG
CTGGCGCCTATATCGCCGACATCACCGATGGGGAAGATCGGGCTCGCCACTTCGGGCTCATGAGCGCTTGTTTCGGCGTG
GGTATGGTGGCAGGCCCCGTGGCCGGGGGACTGTTGGGCGCCATCTCCTTGCATGCACCATTCCTTGCGGCGGCGGTGC
TCAACGGCCTCAACCTACTACTGGGCTGCTTCCTAATGCAGGAGTCGCATAAGGGAGAGCGTCGAGATCCCGGACACCATC
GAATGGTGCAAAACCTTTCGCGGTATGGCATGATAGCGCCCGGAAGAGAGTCAATTGAGGGTGGTGAATGTGGCTAGTTTT
CAATCATTTGGGATACCAGGACAGCTGGAAGTCATCAAAAAAGCACTTGATCACGTGCGAGTCGGTGTGGTAATTACAGATC
CCGCACTTGAAGATAATCCTATTGTCTACGTAAATCAAGGCTTTGTTCAAATGACCGGCTACGAGACCGAGGAAATTTTAGG
AAAGAACTGTCGCTTCTTACAGGGGAAACACACAGATCCTGCAGAAGTGGACAACATCAGAACCGCTTTACAAAATAAAGAA
CCGGTCACCGTTCAGATCCAAAACTACAAAAAAGACGGAACGATGTTCTGGAATGAATTAAATATTGATCCAATGGAAATAGA
GGATAAAACGTATTTTGTCGGAATTCAGAATGATATCACCGAGCACCAGCAGACCCAGGCGCGCCTCCAGGAACTGCAATC
CGAGCTCGTCCACGTCTCCAGGCTGAGCGCCATGGGCGAAATGGCGTCCGCGCTCGCGCACGAGCTCAACCAGCCGCTG
GCGGCGATCAGCAACTACATGAAGGGCTCGCGGCGGCTGCTTGCCGGCAGCAGTGATCCGAACACACCGAAGGTCGAAA
GCGCCCTGGACCGCGCCGCCGAGCAGGCGCTGCGCGCCGGCCAGATCATCCGGCGCCTGCGCGACTTCGTTGCCCGCG
GCGAATCGGAGAAGCGGGTCGAGAGTCTCTCCAAGCTGATCGAGGAGGCCGGCGCGCTCGGGCTTGCCGGCGCGCGCG
AGCAGAACGTGCAGCTCCGCTTCAGTCTCGATCCGGGCGCCGATCTCGTTCTCGCCGACCGGGTGCAGATCCAGCAGGTC
CTGGTCAACCTGTTCCGCAACGCGCTGGAAGCGATGGCTCAGTCGCAGCGACGCGAGCTCGTCGTCACCAACACCCCCGC
CGCCGACGACATGATCGAGGTCGAAGTGTCCGACACCGGCAGCGGTTTCCAGGACGACGTCATTCCGAACCTGTTTCAGA
CTTTCTTCACCACCAAGGACACCGGCATGGGCGTGGGACTGTCCATCAGCCGCTCGATCATCGAAGCTCACGGCGGGCGC
ATGTGGGCCGAGAGCAACGCATCGGGCGGGGCGACCTTCCGCTTCACCCTCCCGGCAGCCGACGAGATGATAGGAGGTC
TAGCATGACGACCAAGGGACATATCTACGTCATCGACGACGACGCGGCGATGCGGGATTCGCTGAATTTCCTGCTGGATTC
TGCCGGCTTCGGCGTCACGCTGTTTGACGACGCGCAAGCCTTTCTCGACGCCCTGCCGGGTCTCTCCTTCGGCTGCGTCG
TCTCCGACGTGCGCATGCCGGGCCTTGACGGCATCGAGCTGTTGAAGCGGATGAAGGCGCAGCAAAGCCCCTTTCCGATC
CTCATCATGACCGGTCACGGCGACGTGCCGCTCGCGGTCGAGGCGATGAAGTTAGGGGCGGTGGACTTTCTGGAAAAGCC
TTTCGAGGACGACCGCCTCACCGCCATGATCGAATCGGCGATCCGCCAGGCCGAGCCGGCCGCCAAGAGCGAGGCCGTC
GCGCAGGATATCGCCGCCCGCGTCGCCTCGTTGAGCCCCAGGGAGCGCCAGGTCATGGAAGGGCTGATCGCCGGCCTTT
CCAACAAGCTGATCGCCCGCGAGTACGACATCAGCCCGCGCACCATCGAGGTGTATCGGGCCAACGTCATGACCAAGATG
CAGGCCAACAGCCTTTCGGAGCTGGTTCGCCTCGCGATGCGCGCCGGCATGCTCAACGATTGACAATTGATGTAAGTTAGC
TCACTCATTAGGCACCGGGATCTCGACCGATGCCCTTGAGAGCCTTCAACCCAGTCAGCTCCTTCCGGTGGGCGCGGGGC
ATGACTATCGTCGCCGCACTTATGACTGTCTTCTTTATCATGCAACTCGTAGGACAGGTGCCGGCAGCGCTCTGGGTCATTT
TCGGCGAGGACCGCTTTCGCTGGAGCGCGACGATGATCGGCCTGTCGCTTGCGGTATTCGGAATCTTGCACGCCCTCGCT
CAAGCCTTCGTCACTGGTCCCGCCACCAAACGTTTCGGCGAGAAGCAGGCCATTATCGCCGGCATGGCGGCCCCACGGGT
GCGCATGATCGTGCTCCTGTCGTTGAGGACCCGGCTAGGCTGGCGGGGTTGCCTTACTGGTTAGCAGAATGAATCACCGAT
ACGCGAGCGAACGTGAAGCGACTGCTGCTGCAAAACGTCTGCGACCTGAGCAACAACATGAATGGTCTTCGGTTTCCGTGT
TTCGTAAAGTCTGGAAACGCGGAAGTCAGCGCCCTGCACCATTATGTTCCGGATCTGCATCGCAGGATGCTGCTGGCTACC
CTGTGGAACACCTACATCTGTATTAACGAAGCGCTGGCATTGACCCTGAGTGATTTTTCTCTGGTCCCGCCGCATCCATACC
GCCAGTTGTTTACCCTCACAACGTTCCAGTAACCGGGCATGTTCATCATCAGTAACCCGTATCGTGAGCATCCTCTCTCGTT
TCATCGGTATCATTACCCCCATGAACAGAAATCCCCCTTACACGGAGGCATCAGTGACCAAACAGGAAAAAACCGCCCTTAA
CATGGCCCGCTTTATCAGAAGCCAGACATTAACGCTTCTGGAGAAACTCAACGAGCTGGACGCGGATGAACAGGCAGACAT
CTGTGAATCGCTTCACGACCACGCTGATGAGCTTTACCGCAGCTGCCTCGCGCGTTTCGGTGATGACGGTGAAAACCTCTG
ACACATGCAGCTCCCGGAGACGGTCACAGCTTGTCTGTAAGCGGATGCCGGGAGCAGACAAGCCCGTCAGGGCGCGTCA
GCGGGTGTTGGCGGGTGTCGGGGCGCAGCCATGACCCAGTCACGTAGCGATAGCGGAGTGTATACTGGCTTAACTATGCG
GCATCAGAGCAGATTGTACTGAGAGTGCACCATTGCGGTGTGAAATACCGCACAGATGCGTAAGGAGAAAATACCGCATCA
GGCGCTCTTCCGCTTCCTCGCTCACTGACTCGCTGCGCTCGGTCGTTCGGCTGCGGCGAGCGGTATCAGCTCACTCAAAG
GCGGTAATACGGTTATCCACAGAATCAGGGGATAACGCAGGAAAGAACATGTGAGCAAAAGGCCAGCAAAAGGCCAGGAA
CCGTAAAAAGGCCGCGTTGCTGGCGTTTTTCCATAGGCTCCGCCCCCCTGACGAGCATCACAAAAATCGACGCTCAAGTCA
GAGGTGGCGAAACCCGACAGGACTATAAAGATACCAGGCGTTTCCCCCTGGAAGCTCCCTCGTGCGCTCTCCTGTTCCGAC
CCTGCCGCTTACCGGATACCTGTCCGCCTTTCTCCCTTCGGGAAGCGTGGCGCTTTCTCATAGCTCACGCTGTAGGTATCT
CAGTTCGGTGTAGGTCGTTCGCTCCAAGCTGGGCTGTGTGCACGAACCCCCCGTTCAGCCCGACCGCTGCGCCTTATCCG
GTAACTATCGTCTTGAGTCCAACCCGGTAAGACACGACTTATCGCCACTGGCAGCAGCCACTGGTAACAGGATTAGCAGAG
CGAGGTATGTAGGCGGTGCTACAGAGTTCTTGAAGTGGTGGCCTAACTACGGCTACACTAGAAGGACAGTATTTGGTATCT

```
GCGCTCTGCTGAAGCCAGTTACCTTCGGAAAAAGAGTTGGTAGCTCTTGATCCGGCAAACAAACCACCGCTGGTAGCGGTG
GTTTTTTTGTTTGCAAGCAGCAGATTACGCGCAGAAAAAAAGGATCTCAAGAAGATCCTTTGATCTTTTCTACGGGGTCTGAC
GCTCAGTGGAACGAAAACTCACGTTAAGGGATTTTGGTCATGAACAATAAAACCGCAAGCGAACCGGAATTGCCAGCTGGG
GCGCCCTCTGGTAAggttgggaagccctgcaaAGTAAACTGGATGGCTTTCTTGCCGCCAAGGATCTGATGGCGCAGGGGATCAAG
ATCTGATCAAGAGACAGGATGAGGATCGTTTCGTATGAGGGAAGCGGTGATCGCCGAAGTATCGACTCAACTATCAGAGGT
AGTTGGCGTCATCGAGCGCCATCTCGAACCGACGTTGCTGGCCGTACATTTGTACGGCTCCGCAGTGGATGGCGGCCTGA
AGCCACACAGTGATATTGATTTGCTGGTTACGGTGACCGTAAGGCTTGATGAAACAACGCGGCGAGCTTTGATCAACGACCT
TTTGGAAACTTCGGCTTCCCCTGGAGAGAGCGAGATTCTCCGCGCTGTAGAAGTCACCATTGTTGTGCACGACGACATCATT
CCGTGGCGTTATCCAGCTAAGCGCGAACTGCAATTTGGAGAATGGCAGCGCAATGACATTCTTGCAGGTATCTTCGAGCCA
GCCACGATCGACATTGATCTGGCTATCTTGCTGACAAAAGCAAGAGAACATAGCGTTGCCTTGGTAGGTCCAGCGGCGGAG
GAACTCTTTGATCCGGTTCCTGAACAGGATCTATTTGAGGCGCTAAATGAAACCTTAACGCTATGGAACTCGCCGCCCGACT
GGGCTGGCGATGAGCGAAATGTAGTGCTTACGTTGTCCCGCATTTGGTACAGCGCAGTAACCGGCAAAATCGCGCCGAAG
GATGTCGCTGCCGACTGGGCAATGGAGCGCCTGCCGGCCCAGTATCAGCCCGTCATACTTGAAGCTAGACAGGCTTATCTT
GGACAAGAAGAAGATCGCTTGGCCTCGCGCGCAGATCAGTTGGAAGAATTTGTCCACTACGTGAAAGGCGAGATCACCAAG
GTAGTCGGCAAATAAGAGCTCGCTTGGACTCCTGTTGATAGATCCAGTAATGACCTCAGAACTCCATCTGGATTTGTTCAGA
ACGCTCGGTTGCCGCCGGGCGTTTTTTATTGGTGAGAATCCAAGCAGTGCCACCTGAAATTGTAAACGTTAATATTTTGTTAA
AATTCGCGTTAAATTTTTGTTAAATCAGCTCATTTTTTAACCAATAGACCGAAATCGGCAAAATCCCTTATAAATCAAAAGAATA
GACCGAGATAGGGTTGAGTGTTGTTCCAGTTTGGAACAAGAGTCCACTATTAAAGAACGTGGACTCCAACGTCAAAGGGCG
AAAAACCGTCTATCAGGGCGATGGCCCACTACGTGAACCATCACCCTAATCAAGTTTTTTGGGGTCGAGGTGCCGTAAAGC
ACTAAATCGGAACCCTAAAGGGAGCCCCCGATTTAGAGCTTGACGGGGAAAGCCGGCGAACGTGGCGAGAAAGGAAGGGA
AGAAAGCGAAAGGAGCGGGCGCTAGGGCGCTGGCAAGTGTAGCGGTCACGCTGCGCGTAACCACCACACCCGCCGCGCT
TAATGCGCCGCTACAGGGCGCGTCCCATTCGCCA
```

References

1. Zhao F, Chavez MS, Naughton KL, Niman CM, Atkinson JT, Gralnick JA, El-Naggar MY, Boedicker JQ. 2022. Light-induced patterning of electroactive bacterial biofilms. *ACS Synth Biol* 11:2327–2338.
2. Zhao F, Niman CM, Ostovar G, Chavez MS, Atkinson JT, Bonis BM, Gralnick JA, El-Naggar MY, Boedicker JQ. 2024. Red-light-induced genetic system for control of extracellular electron transfer. *ACS Synth Biol* 13:1467–1476.
